# All-Cause Mortality Risk in National Prostate Cancer Cohort: An Impact of Population-Based Prostate Cancer Screening

**DOI:** 10.3390/jcm10112459

**Published:** 2021-06-01

**Authors:** Ausvydas Patasius, Giedre Smailyte

**Affiliations:** 1 Laboratory of Cancer Epidemiology, National Cancer Institute, LT-08406 Vilnius, Lithuania; giedre.smailyte@nvi.lt; 2 Department of Public Health, Institute of Health Sciences, Faculty of Medicine, Vilnius University, LT-03101 Vilnius, Lithuania

**Keywords:** prostate cancer, screening, mortality risk, cause-specific mortality, all-cause mortality

## Abstract

The aim of this study is to evaluate all-cause mortality risk differences before and during prostate cancer screening, with a profound focus on the differences between screened and not-screened patient groups. Prostate cancer cases diagnosed between 1998 and 2016 were identified from the population-based Lithuanian Cancer Registry and linked with screening status in the National Health Insurance Fund database. The analysis was stratified by a period of diagnosis and screening status. Standardized mortality ratios (SMRs) were used to assess all-cause and cause-specific mortality risk. The SMRs were calculated by dividing the observed number of deaths among prostate cancer patients by the expected number of deaths from the general population. All-cause SMR (1.45 (95% CI 1.42–1.48)) in the pre-screening period was higher compared to the screening period (SMR = 1.17 (95% CI 1.15–1.19)). An increased all-cause mortality risk among prostate cancer patients was observed in the not-screened patient population (SMR = 1.76 (95% CI 1.71–1.82)), while all-cause mortality risk in the screened patient population was similar to the general population (SMR = 1.00 (95% CI 0.97–1.02)). Screened patients with localized stage of disease had lower all-cause mortality risk than the general population (SMR = 0.72 (95% CI 0.70–0.75)). In conclusion, men with prostate cancer in Lithuania had excess all-cause mortality risk compared to the general population. The all-cause mortality risk among screened patients was not higher than expected.

## 1. Introduction

The main factor determining the incidence of prostate cancer is the utilization of prostate specific antigen (PSA) test in clinical practice and public health interventions [1]. Despite the wide use of PSA testing in many countries, the benefits of PSA-based screening remain debatable [2]. The best indicator for success against cancer is a decreasing mortality rate in the population [3]. European randomized study of screening for Prostate Cancer (ERSPC) based on the PSA test showed 20% significant reduction in prostate cancer mortality in the screened patient arm compared to not-screened patient arm. Although the ERSPC trial has demonstrated a reduction in cause-specific mortality, a reduction in all-cause mortality was not demonstrated relative to comparing higher risk groups with lower risk groups [4]. In the study from The Prostate, Lung, Colorectal and Ovarian (PLCO) Cancer Screening Trial, higher other and all-cause mortality risk was observed in the high risk prostate cancer group [5]. The trial presupposes that a large sample size in a trial is required in order to demonstrate a significant reduction in all-cause mortality. Disease-specific mortality is biased in favor of screening and all-cause mortality is a more exact endpoint than disease-specific mortality in cancer screening trials [6]. Increased detection of cancer in the screened patients group presupposes a greater likelihood of a death being classified as caused by cancer in this group which leads to ascertainment or so called “sticky diagnosis” bias [7,8]. All-cause mortality is often used as an outcome measure to study the effects of different interventions on the course of prostate cancer in contemporary register-based prostate cancer studies [9]; however, only five studies from the USA, Europe, UK, Sweden, and Finland reported their results [10,11,12].

Lithuania has operated the national Early Prostate Cancer Detection Program (EPCDP) since 2006 [13]. This program works in the setting of nationwide PSA test-based screening and 70% of men aged 50 years to 74 years have participated in the prostate cancer program at least once in the first 10 years of the screening. During the screening period, prostate cancer incidence exhibited a dramatic increase in incidence and reached 279.33 cases per 100,000 (from 69.32 cases per 100,000 in 2000, European standard), with slowly decreasing prostate cancer mortality since 2007 [14]. However, all-cause and other cause mortality risk differences after implementation of prostate screening in Lithuania have never been investigated.

The purpose of this study is to evaluate all-cause and other cause mortality risk differences before and during prostate cancer screening, with a profound focus on all-cause mortality risk differences between screened and not-screened patient groups.

## 2. Materials and Methods

Cause-specific and all-cause mortality risk among patients with prostate cancer in Lithuania were evaluated using a retrospective cohort study design. Primary prostate cancer cases diagnosed between 1998 and 2016 were identified from the population-based Lithuanian Cancer Registry, which covers the entire population (3 million individuals in 2011) of the country and has been in operation since 1984. Available data for this analysis included age, date of diagnosis, date, and cause of death. Underlying causes of death were obtained from death certificates. Screening status (participated at least once in the screening program) was received from the National Health Insurance Fund (NHIF).

The study cohort was divided into two periods: Patients diagnosed with prostate cancer before the official start date of prostate cancer screening 1 January 2006 were assigned to the group “pre-screening”, and patients diagnosed after 1 January 2006 assigned to group the “screening”. In further mortality risk analysis, the screening group was further divided into two groups by screening history status which are “screened” and “not-screened”. The definition of what falls under “not-screened” is an individual who had been diagnosed with prostate cancer but has not participated in the Early Prostate Cancer Detection Program in Lithuania.

Person-years were computed from the date of cancer diagnosis to the first of the following events: death, emigration, or end of the follow-up (31 December 2016). Standardized mortality ratios (SMRs) were calculated by dividing the observed number of deaths among prostate cancer patients by the expected number of deaths estimated from the general population of men, with 95% confidence intervals (CIs). Indirect standardization was used to calculate the expected number of deaths for the study population. Expected numbers were calculated as the multiplication of the exact person—years under observation in the cohort by calendar year—and 5-year-age-groups-specific national cause of death rates [15].

For stage group-specific standardized mortality ratios (SMRs) analysis, we determined three prostate cancer groups by cancer stage and TNM: localized (T1-T2N0M0 or stage I–II), advanced (T3-T4N0M0 or stage III), distant (any T, N1 or M1 or stage IV), and unknown stage. A χ^2^-test for trend was performed to evaluate all-cause mortality risk among prostate cancer patients over stage groups [16].

All statistical analyses were carried out using STATA 15 statistical software (StataCorp LLC. 2020 rev. Stata Statistical Software: Release 15.0., College Station, TX, USA). The study was conducted following the Declaration of Helsinki and the protocol was approved by the Vilnius Regional Biomedical Research Ethics Committee with the approval number 158200-16-879-388 on 28 November 2016. Data analyzed were based on record-linkage information and, as such, the Ethics Committee waived the need for informed consent of the study subject.

## 3. Results

In the Table 1 are the presented characteristics of the study groups. In part A are the described patient cohorts by period. The mean population age before screening was 71.51 years and it was higher compared to the prostate cancer population during the screening —67.17 years. In part B demonstrates patient cohorts by screening status during the screening period. Not-screened prostate cancer patients were older than screened (76.26 vs. 65.47 years). All-cause mortality risk before and during the screening period (Table 2, part A) among prostate cancer patients was higher compared to the general population, however, during screening period all-cause mortality risk was lower than in the pre-screening period (SMR = 1.45 (95% CI 1.42–1.48) vs. SMR = 1.17 (95% CI 1.15–1.19)). An increased all-cause mortality risk among prostate cancer patients (Table 2, part B) was observed in the not-screened patient population (SMR = 1.76 (95% CI 1.71–1.82)) while all-cause mortality risk in screened patient population was similar to general population (SMR = 1.00 (95% CI 0.97–1.02)).

Stage group-specific standardized mortality ratio (Table 3) from all-causes among men with prostate cancer showed a higher risk of death for patients that were locally advanced and metastatic as well as for patients with the unknown stage of disease and risk increased with increasing dissemination (*p* = <0.0001) among the not-screened and screened patient cohort. For persons with the localized disease in not-screened cohort, risk of death was similar to the general population (SMR = 0.99 (95% CI 0.92–1.05)) while for the screened person cohort it was significantly lower (SMR = 0.72 (95% CI 0.70–0.75)).

Supplementary materials Tables S1 and S2 show standardized mortality ratios for specific causes of death. Increased mortality risk for malignant neoplasms was found (Supplementary Table S1). During the pre-screening period, mortality risk was increased for bladder cancer, colorectal neoplasms, prostate, and kidney and renal pelvis cancer although the cancer risk increase was insignificant for colorectal neoplasms and kidney cancer. During the screening period, an increased mortality ratio was observed for kidney and renal pelvis cancer, colorectal neoplasms, prostate, and bladder cancer although the risk increase for bladder cancer was insignificant.

During the screening period, among the screened and not-screened patients, standardized mortality ratios for specific causes of death were increased for malignant neoplasms: colorectal, kidney, renal and pelvis, and bladder cancers among the not-screened patients; and for bladder cancer among screened patients (Supplementary Table S2). SMR’s for the diseases of the circulatory system was not higher than expected from the general population during the whole study period and in different screening status groups.

## 4. Discussion

This national cohort study involved 48,819 men with prostate cancer and was followed-up for almost 250,000 person-years and with over 19,000 deaths. Mortality risks were analyzed from 1998 to 2016. The large study cohort allowed us to investigate a wide range of mortality outcomes and compare them to the general population.

The study showed that people with prostate cancer had a 28% increased risk of death from all causes. Higher mortality risk was observed for patients before prostate cancer screening. All-cause mortality for prostate cancer patients was higher among not-screened patients, while screened patient mortality risk was similar to the general population. Stage group-specific all-cause mortality risk increased with disease severity.

Prostate cancer incidence in Lithuania is often compared with the incidence in the United States of America, where opportunistic PSA screening played a crucial role in prostate cancer diagnostics. Incidence changes in Lithuania in 2007 mimicked prostate cancer incidence changes in the late 1980s and early 1990s in the United States. There was a rapid incidence peak in Lithuania after the beginning of the screening program, followed by a decrease thereafter. It was caused by a so-called “backlog” of prevalent cancers that had accumulated as a result of previous years’ incidence although mortality changes between the United States and Lithuania were different. In the United States there was an observed increase in prostate cancer mortality from the initiation of PSA based prostate screening, which is likely caused by the labelling of prostate cancer diagnosis to death certificates for older men population as opposed to true increases. These changes were followed by a mortality decrease by 37% from baseline prostate cancer mortality level [6]. In Lithuania, there was observed increasing mortality trend from 1985 to 2006 by 3.6% annually and decreasing mortality trend from 2006 by 1.4% annually [14].

All-cause mortality risk among prostate cancer patients was analyzed in studies from Sweden, the USA, Europe (ERSPC), and Finland. In the study from Finland, significant all-cause mortality decreases over the period from 1985 to 2009 and the mortality risk reduction among patients with the localized disease was observed [12]. The study from Sweden compared risk-specific prostate cancer groups with prostate cancer-free control male group [11]. They have found that all-cause mortality was lower in men with low-risk prostate cancer compared with the corresponding prostate cancer-free men. Investigators from the ERSPC trial, comparing different risk groups, have found that prostate cancer screening attendees have lower all-cause mortality rate and a higher probability of a prostate cancer diagnosis than non-attendees although they stated that correction for attendance in screening status is important if the calculation of all-cause mortality risk is to be used in conjunction with disease-specific mortality analysis [4]. The PLCO trial in 2020 reported results of other cause and all-cause mortality risk among prostate cancer patients who participated in the PLCO trial. They have found that other cause survival was higher in lower risk disease compared to higher risk disease although results were statistically insignificant [5].

In the study from Sweden [11] comparing risk-stratified prostate cancer groups with controls, higher cardiovascular mortality risk was observed. It is well known, that one of the treatment modalities—androgen deprivation therapy—which is the treatment keystone of metastatic prostate cancer, has a causal relationship with increased mortality risk from cardiovascular diseases (CVD) [17,18]. In our study, during the pre-screening period and during screening, there was an observed lower mortality risk from diseases of the circulatory system than in the general population. Not-increased mortality risk from diseases of the circulatory system was found by comparing screened and not-screened patient cohorts. These findings differ from findings in other study and this could be explained by the high prevalence of cardiovascular diseases in the population of men in Lithuania [19].

Increased mortality risk due to kidney cancer, bladder cancer, and colorectal cancer was observed during the study period. Patients who are participating in the screening program often tend to be more aware of the benefits of health check-ups and they often undergo additional medical examination [20]. During the diagnostic process of prostate cancer, various imaging modalities are used and, therefore, patients could be incidentally diagnosed with kidney cancer or colorectal cancer [21,22,23,24]. Since those patients have worsened survival outcomes compared to prostate cancer alone, the more likely cause of death is different than for prostate cancer alone. Increased SMR for bladder and colorectal cancer among not-screened persons could be linked to a greater likelihood of second primary cancer due to pelvic radiotherapy, which is used as one of the modalities to treat advanced prostate cancer [25].

In the comparison of two different cohorts of prostate cancer patients (screened and not-screened), the benefit of screening can be observed. Even stage group-specific mortality risk analysis showed higher mortality rates in the not-screened patient cohort. More surprisingly, among screened patients with the localized stage of the disease there was an observed lower all-cause mortality risk compared to the general population. The so called “stage migration” was described in our previous studies [13,26] and the biggest proportion in our population-based screening cohort was localized disease. Therefore, we could assume that lower mortality risk in the screened patient population is mainly caused by earlier diagnosis. The benefit of the treatment of localized disease is seen in a large study from Sweden [27] that combined radical prostatectomy, radiation therapy, and active surveillance where surgery reduced the risk of death due to prostate cancer by 44% and all-cause mortality by 12.7%. In the study from Finland, benefits from early diagnostics were observed as well and the SMR’s for localized disease became similar to the general Finnish male population since the introduction of PSA; furthermore, since the early 2000s, the SMR was lower compared to the SMR in the Finnish male population [12].

Higher stage-specific SMR rates in the not-screened patient cohort might be a reflection of social inequalities in men with prostate cancer population [28]. In Lithuania, age-adjusted incidence rate ratios (IRR) were reported to be higher among men with higher education and a lower risk among men with secondary and lower than secondary education (IRR = 0.70 (95% CI 0.64–0.76) and IRR = 0.49 (95% CI 0.46–0.53)) [29]. Mortality rate ratio comparing secondary and lower than secondary levels of education to prostate cancer patients with higher education was higher in lower than the secondary level of education group (MRR = 1.40 (95% CI 1.13–1.72)). It is worth mentioning that opportunistic testing brings social inequalities as well when the more affluent can more readily obtain the benefits of testing than the less affluent.

Our study has limitations and strengths. The large proportion of men with unknown prostate cancer stage and bias encountered with SMR calculation where the true relative risk can be underestimated for relatively common diseases somewhat limited our analysis; however, it is unlikely to have affected the main conclusions drawn. Lower mortality rates in the screened patient group might be affected by earlier diagnosis and lead-time bias. The lack of clinical data regarding diagnostic modalities used and treatments of prostate cancer, which have largely evolved during study period, somewhat limited our study. The strengths of this study are the analyses based on real-life data, which covers a large national prostate cancer patient cohort, in line with a unique experience from ongoing population-based prostate cancer screening program and long follow-up of patients. Despite that, randomized controlled trials provide the highest level of evidence and analyses of real-world data from population-based registries provide unique information for the management of the effects of diagnosis at the population level.

## 5. Conclusions

In conclusion, men with prostate cancer in Lithuania had excess mortality risk compared to the general population. Population-based prostate cancer screening in Lithuania resulted in a mortality risk decrease in the time frame and mortality risk among screened patients was similar to that expected from the general population. Prostate cancer screening program participants with localized disease experienced mortality risk reduction.

## Figures and Tables

**Table 1:** Study group characteristics of men diagnosed with prostate cancer during pre-screening and screening period (A) and of screened and not-screened persons (B).

A	Pre-Screening (1998–2005)	Screening (2006–2016)
Patients diagnosed with prostate cancer (%) (N ^1^)	11,401	37,418
Median age at prostate cancer diagnosis (IQR ^2^), years.	72 (66; 77)	67 (61; 73)
Mean follow-up time (IQR), days.	2076 (613; 4222)	1728 (859; 2802)
Number of deaths (%)	8782 (77.03)	11,079 (29.61)
**B**	**Not-Screened**	**Screened**
Patients diagnosed with prostate cancer (%) (N ^1^)	5886	31,532
Median age at prostate cancer diagnosis (IQR ^2^), years.	78 (73; 82)	66 (60; 71)
Mean follow-up time (IQR), days.	1093 (261; 2377)	1811 (983; 2852)
Number of deaths (%)	3805 (64.64)	7274 (23.06)

^1^ Number; ^2^ IQR—interquartile range.

**Table 2:** Standardized all-cause mortality ratios during pre-screening and screening period (A) and of screened and not-screened persons (B).

	Obs ^1^	Exp ^2^	SMR ^3^	95% CI ^4^		*p* Value ^5^	Obs	Exp	SMR	95% CI		*p*-Value ^2^
**A**	**Pre-screening (1998–2006)**						**Screening (2006–2016)**					
All-causes	8782	6075.12	1.45	1.42	1.48	<0.001	11,079	9461.78	1.17	1.15	1.19	<0.001
**B**	**Not-Screened**						**Screened**					
All-causes	3805	2157.04	1.76	1.71	1.82	<0.001	7274	7304.74	1.00	0.97	1.02	<0.001

^1^ Obs, observed; ^2^ Exp, expected; ^3^ SMR, standardized mortality ratio; ^4^ CI, confidence interval; ^5^ Chi-square test.

**Table 3:** Stage-specific standardized mortality ratios of screened and not-screened persons.

	Not-Screened						Screened					
Stage Group	Obs	Exp	SMR	95% CI		*p*-Value ^2^	Obs	Exp	SMR	95% CI		*p*-Value ^2^
Overall	3805	2157.04	1.76	1.71	1.82	<0.001	7274	7304.74	1.00	0.97	1.02	<0.001
Localized	886	897.05	0.99	0.92	1.05	0.712	2878	3974.42	0.72	0.70	0.75	<0.001
Locally-advanced	1076	779.70	1.38	1.30	1.46	<0.001	1979	1607.36	1.23	1.18	1.29	<0.001
Distant	588	107.71	5.46	5.04	5.92	<0.001	473	114.59	4.13	3.77	4.52	<0.001
Unknown	1255	372.58	3.37	3.19	3.56	<0.001	1944	1608.38	1.21	1.16	1.26	<0.001
	*p*^1^ < 0.0001						*p*^1^ < 0.0001					

^1^ χ^2^ test for trend, ^2^ Chi-square test.

## Data Availability

The data presented in this study are available on request from the corresponding author. The data are not publicly available due to ethical restrictions.

## Supplementary Materials

The following are available online at https://www.mdpi.com/article/10.3390/jcm10112459/s1, Table S1: Cause-specific standardized mortality ratios of men diagnosed with prostate cancer during pre-screening and screening period, Table S2: Cause-specific standardized mortality ratios of screened and not-screened persons.
