# Supplementary material for: All-Cause Mortality Risk in National Prostate Cancer Cohort: An Impact of Population-Based Prostate Cancer Screening
Source: J Clin Med. 2021 Jun 1;10(11):2459. doi: 10.3390/jcm10112459 (PMC8199572; doi:10.3390/jcm10112459)
Supplement: Supplementary file 1 [file jcm-10-02459-s001.zip › jcm-1203485-supplementary.pdf]

Table S1. Cause-specific standardized mortality ratios of men diagnosed with prostate cancer during pre-screening and screening period.

	**Pre-Screening (1998–2005)**						**Screening (2006–2016)**					
**Diagnosis (ICD-10 Code)**	**Obs** [1]	**Exp** [2]	**SMR** [3]	**95% CI** [4]		***p* Value** [5]	**Obs**	**Exp**	**SMR**	**95% CI**		***p*-Value**[5]
Certain infectious and parasitic diseases (A00-B99)	28	50	0.56	0.39	0.82	0.002	77	125.38	0.61	0.49	0.77	<0.001
Malignant neoplasms (C00-C97)	5200	1190	4.37	4.25	4.49	<0.001	5795	2139.48	2.71	2.64	2.78	<0.001
Colorectal neoplasms (C18-C21)	156	148	1.05	0.90	1.23	0.518	264	233.37	1.13	1.00	1.28	0.050
Prostate cancer (C61)	4350	228	19.04	18.48	19.61	<0.001	4045	335.27	12.06	11.70	12.44	<0.001
Kidney and renal pelvis (C64-C65)	54	42	1.28	0.98	1.66	0.064	155	87.06	1.78	1.52	2.08	<0.001
Bladder (C67)	89	64	1.39	1.13	1.71	0.002	189	173.61	1.09	0.94	1.26	0.243
Endocrine, nutritional, and metabolic diseases (E00-E88)	25	34	0.74	0.50	1.09	0.122	47	64.82	0.73	0.54	0.97	0.027
Diabetes mellitus (E10-E14)	21	32	0.67	0.43	1.02	0.052	39	60.32	0.65	0.47	0.88	0.006
Mental and behavioral disorders (F01-F99)	4	9	0.44	0.17	1.18	0.096	12	18.70	0.64	0.36	1.13	0.121
Diseases of the nervous system (G00-G98)	41	45	0.91	0.67	1.24	0.551	81	53.17	1.52	1.23	1.89	<0.001
Diseases of the circulatory system (I00-I99)	2717	3753	0.72	0.70	0.75	<0.001	3848	5178.26	0.74	0.72	0.77	<0.001
Diseases of the respiratory system (J00-J98)	208	346	0.60	0.52	0.69	<0.001	283	465.70	0.61	0.54	0.68	<0.001
Diseases of the digestive system (K00-K92)	132	178	0.74	0.63	0.88	<0.001	306	388.07	0.79	0.70	0.88	<0.001
Diseases of the genitourinary system (N00-N98)	38	60	0.64	0.46	0.87	0.005	60	81.54	0.74	0.57	0.95	0.017
External causes of mortality (V01-Y98)	103	247	0.42	0.34	0.51	<0.001	360	532.53	0.68	0.61	0.75	<0.001

[1] Obs, observed; [2] Exp, expected; [3] SMR, standardized mortality ratio; [4] CI, confidence interval; [5] Chi-square test.

Table S2. Cause-specific standardized mortality ratios of screened and not-screened persons.

	**Not-Screened Patients**						**Screened Patients**					
**Diagnosis (ICD-10 Code)**	**Obs** [1]	**Exp** [2]	**SMR** [3]	**95% CI** [4]		***p*-Value** [5]	**Obs**	**Exp**	**SMR**	**95% CI**		***p*-Value** [5]
Certain infectious and parasitic diseases (A00-B99)	12	19.87	0.60	0.34	1.06	0.007	65	105.51	0.62	0.48	0.79	<0.001
Malignant neoplasms (C00-C97)	2176	296.01	7.35	7.05	7.67	<0.001	3619	1843.47	1.96	1.90	2.03	<0.001
Colorectal neoplasms (C18-C21)	76	37.54	2.02	1.62	2.54	<0.001	188	195.83	0.96	0.83	1.11	0.576
Prostate cancer (C61)	1804	86.12	20.95	20	21.94	<0.001	2241	249.15	8.99	8.63	9.37	<0.001
Kidney and renal pelvis (C64-C65)	23	9.46	2.43	1.62	3.66	<0.001	65	68.91	0.94	0.74	1.20	0.638
Bladder (C67)	57	18.42	3.09	2.39	4.01	<0.001	132	77.59	1.70	1.43	2.02	<0.001
Endocrine, nutritional, and metabolic diseases (E00-E88)	7	11.66	0.60	0.29	1.26	0.172	40	53.2	0.75	0.55	1.03	0.070
Diabetes mellitus (E10-E14)	6	11.06	0.54	0.24	1.21	0.128	33	49.3	0.67	0.48	0.94	0.020
Mental and behavioral disorders (F01-F99)	1	4.37	0.23	0.03	1.62	0.107	11	14.3	0.77	0.43	1.39	0.382
Diseases of the nervous system (G00-G98)	26	7.94	3.28	2.23	4.81	<0.001	55	45.23	1.22	0.93	1.58	0.146
Diseases of the circulatory system (I00-I99)	1301	1396.11	0.93	0.88	0.98	<0.001	2547	3782.1	0.67	0.65	0.70	<0.001
Diseases of the respiratory system (J00-J98)	92	112.15	0.82	0.67	1.01	0.057	191	353.5	0.54	0.47	0.62	<0.001
Diseases of the digestive system (K00-K92)	76	62.98	1.21	0.96	1.51	0.101	230	325.1	0.71	0.62	0.81	<0.001
Diseases of the genitourinary system (N00-N98)	19	23.36	0.81	0.52	1.27	0.367	41	58.2	0.70	0.52	0.96	0.024
External causes of mortality (V01-Y98)	54	67.66	0.80	0.61	1.04	0.096	306	464.9	0.66	0.59	0.74	<0.001

[1] Obs, observed; [2] Exp, expected; [3] SMR, standardized mortality ratio; [4] CI, confidence interval; [5] Chi-square test.
